# Novel nomogram for predicting risk of early postoperative small bowel obstruction after right colectomy for cancer

**DOI:** 10.1186/s12957-022-02489-2

**Published:** 2022-01-21

**Authors:** Huida Zheng, Yurong Liu, Zhenze Chen, Yafeng Sun, Jianhua Xu

**Affiliations:** Department of Gastrointestinal Surgery, The Second Affiliated Hospital of Fujian Medical University, No. 950 Donghai Street, Fengze District, Quanzhou, 362000 Fujian Province China

**Keywords:** Nomogram, Right colectomy, Early postoperative small bowel obstruction, Risk factors

## Abstract

**Background:**

Early postoperative small bowel obstruction (EPSBO) is a common complication after colorectal cancer surgery. Few studies have specifically studied risk factors for early small bowel obstruction after right colectomy, especially in establishing predictive models. The purpose of the current study was to establish an effective nomogram to predict the incidence of EPSBO after right colectomy.

**Methods:**

The current study retrospectively analyzed data from a total of 424 patients who underwent right colectomy in a local hospital from January 2014 to March 2021. A logistic regression model was used to identify potential risk factors for EPSBO after right colectomy. A nomogram was established by independent risk factors, and the prediction performance of the model was evaluated using an area under the receiver operating characteristic (ROC) curve and calibration chart.

**Results:**

A total of 45 patients (10.6%) developed early small bowel obstruction after right colectomy. Male sex, history of abdominal surgery, open surgery, long operative time, anastomotic leakage, and preoperative albumin were closely related to EPSBO. Analysis of postoperative rehabilitation indices showed that EPSBO remarkably slowed the postoperative rehabilitation speed of patients. Multivariate logistic regression analysis showed that male sex, open surgery, operative time, and anastomotic leakage were independent risk factors (*P* < 0.05), and the operation time had the greatest impact on EPSBO. On the basis of multivariate logistic regression, a nomogram was constructed, which showed moderate accuracy in predicting EPSBO, with a *C*-statistic of 0.716. The calibration chart showed good consistency between the predicted probability and ideal probability.

**Conclusion:**

The current study constructed a nomogram based on the clinical data of patients who underwent right colectomy, which had moderate predictability and could provide reference value for clinicians to evaluate the risk of EPSBO.

## Introduction

Colorectal cancer (CRC) is the third most common malignant tumor worldwide and the second leading cause of cancer-related deaths [1]. Although various treatments for colon cancer have been reported, surgical resection is still the most important treatment [2]. However, there were some studies showed that surgical resection is associated with various complications, with incidence rates ranging between 10% and 37%, which seriously affects the postoperative quality of life and survival time of patients [3]. In particular, early postoperative small bowel obstruction (EPSBO) is a complication of colorectal cancer surgery. The incidence of EPSBO after colorectal cancer surgery was 4.5–12.8% and that the incidence of EPSBO after right hemicolectomy remained high without declining [4, 5]. Previous studies also reported that EPSBO inhibited early oral intake and early ambulation, prolonged postoperative hospital stay, increased cost of hospitalization, and reduced utilization rate of hospital beds [6]. In addition, extension of hospital stay leads to the inability of patients to undergo timely postoperative adjuvant therapy (less than 8 weeks), which reduces the effectiveness of adjuvant therapy in patients with colorectal cancer [7]. There have study also showed that EPSBO was an independent risk factor for secondary adhesive intestinal obstruction [8].

Different surgical methods and surgical ranges for right colon cancer have been used, and these have led to different incidences of postoperative complications, including complete mesocolic excision (CME) and D3 lymph node dissection, standard operation and extended right colectomy, and differences in the medial boundary of lymph node dissection [9–11]. Currently, few studies on risk factors for EPSBO after right hemicolectomy have been reported, especially to establish predictive models. Therefore, there is a need to establish a predictive model of EPSBO after right hemicolectomy and determine controllable risk factors. Few previous studies have explored risk factors for EPSBO after colorectal cancer surgery, including, male sex, operative time, ASA > grade 3, open surgery, emergency surgery, intraoperative blood loss, previous abdominal surgery history, and defunctioning ileostomy [4–6, 12–14]. The purpose of the current study was to construct a nomogram to predict the incidence of EPSBO after right colectomy. This will help clinicians control risk factors in patients with right colectomy and reduce EPSBO incidence, shorten postoperative hospital stay and improve quality of life.

## Methods

### Patients

In total, 424 patients who underwent right colectomy in the Second Affiliated Hospital of Fujian Medical University from January 2014 to March 2021 were retrospectively recruited. EPSBO can be diagnosed if the patient has three or more clinical symptoms such as abdominal pain, abdominal distention, nausea, vomiting, no exhaust, or defecation for more than 24 h within 30 days after colectomy, or signs such as abdominal tenderness, hyperactivity, metal sound, abdominal X-ray, or CT examination indicates small intestinal dilatation and gas-liquid level [4]. The inclusion criteria included patients who underwent right colectomy, which was confirmed as malignant by pathology. The exclusion criteria included a previous history of intraperitoneal radiotherapy, chemotherapy, or inflammatory bowel disease [4, 13]. Recruited study patients were divided into two groups: the EPSBO and no EPSBO groups. All operations were undertaken by experienced gastrointestinal surgeons. The anastomotic method was stapler anastomoses. All patients underwent adequate preoperative evaluation, and there were no significant differences in perioperative management. The current retrospective study was approved by the hospital ethics committee.

### Variables

Variables analyzed as EPSBO risk factors included age, sex, anemia, preoperative obstruction, hypertension, diabetes, BMI, emergency operation, history of abdominal surgery, neoadjuvant therapy, ASA score, type of surgery (standard or extended), surgical approach (open or laparoscopy), blood loss, operative time, tumor diameter, preoperative NLR (neutrophil-lymphocyte ratio), preoperative albumin, pathological T stage, preoperative CEA, pathological N stage, metastasis, anastomotic leakage, and incision infection.

### Statistical analyses

IBM SPSS software (version 22.0) was used for statistical analyses. The results are shown as the mean ± standard error of the mean (s.e.m.) or median (quartile 1, quartile 3). Data conforming to a normal distribution were analyzed using *t* tests (two-tailed), whereas data not conforming to a normal distribution were analyzed using Mann–Whitney rank-sum tests. Fisher’s exact test or Pearson *χ*2 test were used to analyze grade data between groups. Univariate and multivariate logistic regression were used, where variables with *P* < 0.1 in univariate analysis were included in the multivariate model. *P* <0.05 was considered statistically significant.

Forest plots, nomograms, ROC curves and correction curves were drawn using R software (version 4.0.5). Through 1000 bootstrap resamplings, internal verification of the nomogram was completed, and the *C*-statistic/area under the receiver operating characteristic (ROC) curve (AUC) was computed to evaluate the performance of the model. A calibration curve was used to show consistency between the observation frequency and prediction probability.

## Results

### Patients

In total, 424 patients who underwent right hemicolectomy were included in the current study. The patients’ characteristics are summarized in Table 1. The findings showed that EPSBO occurred in 45 patients (10.6%). In addition, male sex (*P* = 0.013), history of abdominal surgery (*P* = 0.026), open surgery (*P* = 0.012), operative time (*P* = 0.002), preoperative albumin (*P* = 0.029), and anastomotic leakage (*P* = 0.024) were significantly associated with EPSBO and were considered risk factors. However, in the current study, ASA ≥ 3, emergency surgery, intraoperative bleeding and preoperative intestinal obstruction were not strongly related to EPSBO (*P* < 0.05).

**Table 1:** Characteristics of patients

Variables	No EPSBO (*n* = 379)	EPSBO (*n* = 45)	***P*** value
Age (years)	65 (57-72)	64 (61-66)	0.466
Gender			0.013
Male	187 (85.8%)	31 (14.2%)	
Female	192 (93.2%)	14 (6.8%)	
Anemia			0.117
Yes	189 (87.1%)	28 (12.9%)	
No	190 (91.8%)	17 (8.2%)	
Preoperative obstruction			0.356
Yes	109 (91.6%)	10 (8.4%)	
No	270 (88.5%)	35 (11.5%)	
Hypertension			0.779
Yes	74 (90.2%)	8 (9.8%)	
No	305 (89.2%)	37 (10.8%)	
Diabetes			0.623
Yes	49 (87.5%)	7 (12.5%)	
No	330 (89.7%)	38 (10.3%)	
BMI (kg/m^2^)			0.356
≤ 25	261 (88.5%)	34 (11.5%)	
> 25	118 (91.5%)	11 (8.5%)	
Emergency operation			0.087
Yes	29 (80.6%)	7 (19.4%)	
No	350 (90.2%)	38 (9.8%)	
History of abdominal surgery			0.026
Yes	47 (81.0%)	11 (19.0%)	
No	332 (90.7%)	34 (9.3%)	
Surgical medial boundary			0.347
SMV	316 (90.0%)	35 (10.0%)	
SMA	63 (86.3%)	10 (13.7%)	
Neoadjuvant therapy			0.288
Yes	8 (80.0%)	2 (20.0%)	
No	371 (89.6%)	43 (10.4%)	
ASA score			0.676
1 or 2	284 (89.0%)	35 (11.0%)	
≥ 3	95 (90.5%)	10 (9.5%)	
Type of surgery			0.365
Standard	269 (90.3%)	29 (9.7%)	
Extended	110 (87.3%)	16 (12.7%)	
Surgical approach			0.012
Open	67 (81.7%)	15 (18.3%)	
Laparoscopy	312 (91.2%)	30 (8.8%)	
Blood loss (mL)	60 (50–100)	50 (50–95)	0.461
Operative time (min)	150 (135–165)	155 (145–172)	0.002
Tumor diameter (cm)	5.0 (4.0–6.5)	4.1 (2.6–7.6)	0.159
Preoperative NLR	2.5 (1.7–3.9)	2.2 (1.5–3.1)	0.059
Preoperative albumin	39.1 (35.1–43.1)	37.2 (33.7–41.0)	0.029
Preoperative CEA	3.9 (2.1–13.1)	4.5 (4.0–6.0)	0.739
Pathological T stage			0.296
T0–T2	37 (82.2%)	8 (17.8%)	
T3–T4	342 (90.2%)	37 (9.8%)	
Pathological N stage			0.670
N0	181 (88.7%)	23 (11.3%)	
N1–N2	198 (90.0%)	22 (10.0%)	
Metastasis			0.768
Yes	29 (87.9.%)	4 (12.1%)	
No	350 (89.5%)	41 (10.5%)	
Anastomotic leakage			0.024
Yes	22 (75.9.%)	7 (24.1%)	
No	357 (90.4%)	38 (9.6%)	
Incision infection			0.979
Yes	50 (89.3%)	6 (10.7%)	
No	329 (89.4%)	39 (10.6%)	

Data are presented as mean ± standard deviation or median (quartile 1, quartile 3)

*Abbreviations*: *BMI* Body mass index, *ASA* American Society of Anesthesiologists, *SMA* Superior mesenteric artery, *SMV* Superior mesenteric vein

### Univariate and multivariate logistic regression analysis

**Fig. 1:**
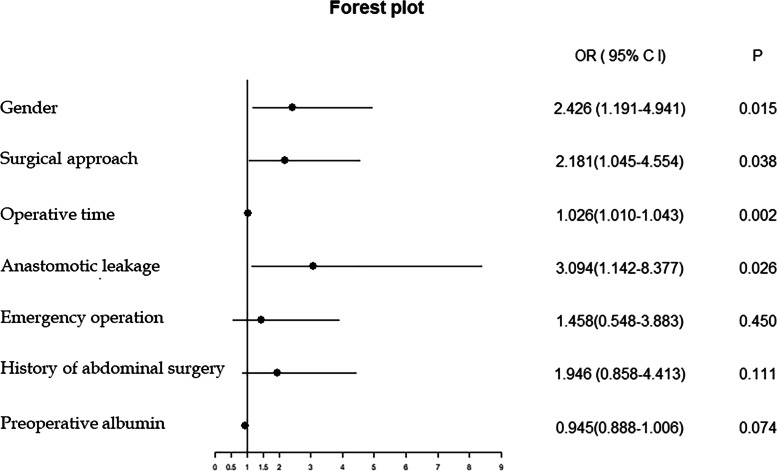
Forest plot

Univariate and multivariate logistic regression analyses were undertaken. The findings, presented in Table 2, showed that male sex, history of abdominal surgery, open surgery, operative time, preoperative albumin, and anastomotic leakage were closely related to EPSBO. The multivariate analysis showed that sex (OR 2.426, 95%CI 1.191–4.941; *P* = 0.015), open surgery (OR 2.181, 95%CI 1.045–4.554; *P* = 0.038), operative time (OR = 1.026,95%CI 1.010–1.043; *P* = 0.002), and anastomotic leakage (OR 3.094,95%CI 1.142–8.377, *P* = 0.026) were independent risk factors for EPSBO. Of note, the operative time had the greatest effect on EPSBO. A forest plot was drawn based on the results of the multivariate regression analysis (Fig. 1).

**Table 2:** Univariate and multivariate analyses for risk factors of EPSBO after right colectomy

		Univariate	Multivariate	
		***P***	***OR*** (95% ***CI)***	***P***
Age (years)		0.552		
Gender	Male or female	0.015	2.426 (1.191–4.941)	0.015
Anemia	Present vs absent	0.120		
Preoperative obstruction	Present vs absent	0.358		
Hypertension	Present vs absent	0.779		
Diabetes	Present vs absent	0.623		
BMI (kg/m2)	> 25 vs ≤ 25	0.358		
Emergency operation	Present vs absent	0.079	1.458 (0.548–3.883)	0.450
History of abdominal surgery	Present vs absent	0.030	1.946 (0.858–4.413)	0.111
Neoadjuvant therapy	Present vs absent	0.341		
ASA score	1 or 2 vs ≥ 3	0.676		
Type of surgery	Standard vs extended	0.366		
Surgical medial boundary	SMV vs SMA	0.349		
Surgical approach	Open vs laparoscopy	0.014	2.181 (1.045–4.554)	0.038
Blood loss (ml)		0.389		
Operative time (min)		< 0.001	1.026 (1.010–1.043)	0.002
Tumor diameter (cm)		0.277		
Preoperative NLR		0.235		
Preoperative albumin		0.038	0.945 (0.888–1.006)	0.074
Preoperative CEA		0.382		
Pathological T stage	T0–T2 vs T3–T4	0.105		
Pathological N stage	N0 vs N1–N2	0.874		
Metastasis	Present vs absent	0.919		
Anastomotic leakage	Present vs absent	0.019	3.094 (1.142–8.377)	0.026
Incision infection	Present vs absent	0.979

### Nomogram for EPSPO

**Fig. 2:**
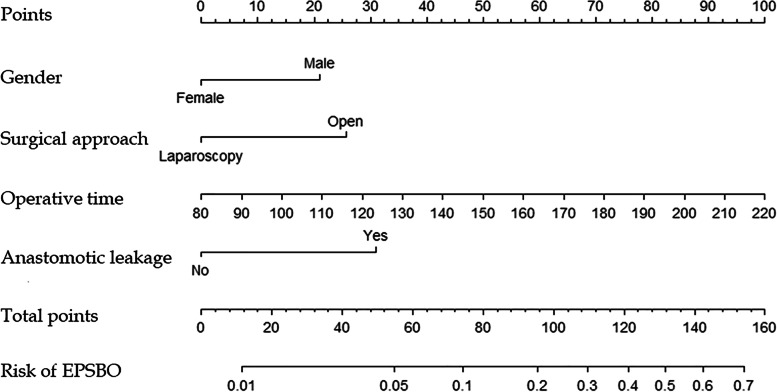
Nomogram for predicting risk of early postoperative small bowel obstruction after right colectomy. Locate male or female on the horizontal axis of gender. Draw a line up to the point axis to determine the number of points toward EPSBO the patient should receive. Repeat this process for other horizontal axes, drawing a straight line to the point axis at a time. Then, calculate the sum of points for all risk factors. Draw a straight line down to find out the risk of EPSBO

**Fig. 3:**
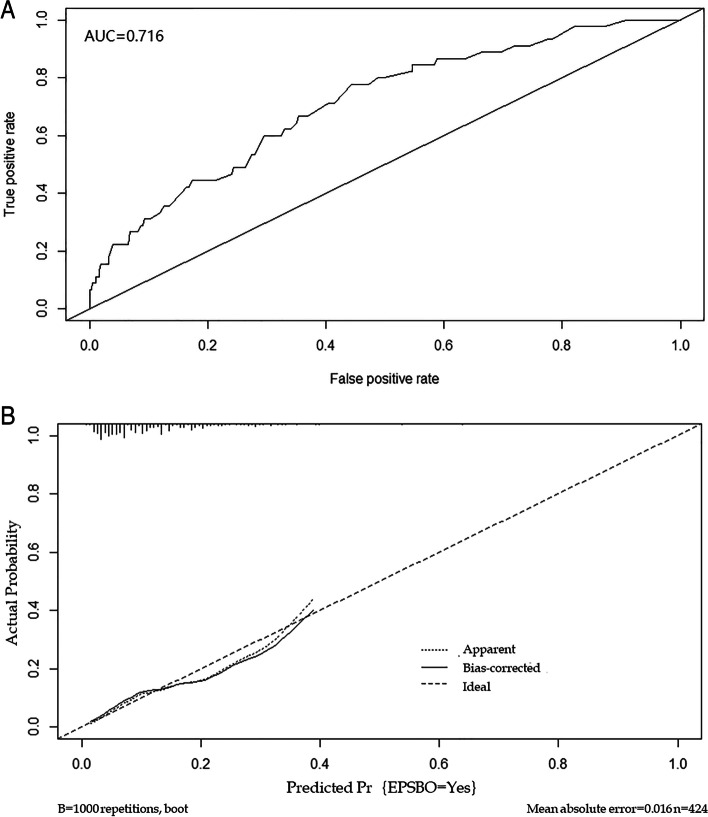
Validation of the nomogram. **A** ROC analysis of EPSBO. The AUC value is 0.716 (95% CI, 0.639–0.794). **B** Calibration plot of the nomogram for EPSBO. The *y*-axis represents the actual probability of EPSBO. The *x*-axis represents the estimated probability, and the ideal line is close to the actual line, indicating a good prediction

A nomogram to predict the risk of EPSBO after right colectomy was constructed based on the results obtained by multivariate logistic regression. The nomogram showed that male sex, open surgery, operative time, and anastomotic leakage were significantly correlated with EPSBO (Fig. 2). Each factor in the nomogram had a scoring standard, with a higher total score indicating a greater risk of EPSBO. For example, a male patient who underwent open surgery with an operative time of 150 min and no anastomotic leakage had a total score of 95 points (20 points for male, 25 points for open surgery, 50 points for operative time, 0 points for anastomotic leakage), leading to an approximately 20% predicted risk of EPSBO. The area under the ROC curve was then computed, which showed that the nomogram had good predictive ability. The AUC value of the model was 0.716 (Fig. 3A), which indicated that the C-statistic value was 0.716 and that the predictive ability of the model for EPSBO risk was medium. The calibration curve showed that the predictive model established in the current study was close to the ideal state, indicating good calibration (Fig. 3B).

### Postoperative characteristics of patients

The postoperative characteristics of EPSBO patients, including time to first ambulation, day of first flatus, day of first meal, drainage tube removal time, and length of hospital stay, are shown in Table 3. In the EPSBO group, the time to first ambulation was significantly longer than those in the non-EPSBO group. EPSBO prolonged the drainage tube removal time, which led to a significantly higher hospitalization time of patients than those without EPSBO. Patients with anastomotic leakage were excluded to reduce the influence of anastomotic leakage on postoperative rehabilitation indicators, and the data were reanalyzed. The findings are shown in Table 4. Significant differences in time to first ambulation (2.0 ± 0.9 vs. 2.6 ± 1.5), length of hospitalization (13.0 ± 4.2 vs. 16.8 ± 5.7) and time to remove drainage tube (9.1 ± 2.5 vs. 11.53 ± 3.6) between the two groups (*P* < 0.001) were observed. The day of first flatus (1.8 ± 1.1 vs. 2.3 ± 1.7) and day of first meal (3.0 ± 1.4 vs 4.1 ± 3.6) in the EPSBO group were longer than those in the non-EPSBO group, although the difference was not significant (*P* = 0.097 and *P* = 0.379). Among EPSBO patients who were included in the current study, two patients needed reoperation, one due to incisional hernia, and the other due to internal hernia (2/45, 4.4%). Fortunately, no patients died due to EPSBO.

**Table 3:** Postoperative characteristics of patients with EPSBO

Variables	No EPSBO (*n* = 379)	EPSBO (*n* = 45)	***P*** value
First ambulation (postoperative day)	2.0 ± 0.9	2.7 ± 1.6	0.004
Day of first meal (postoperative day)	3.0 ± 2.0	4.4 ± 4.3	0.489
Removal of drainage (days)	9.4 ± 3.1	14.4 ± 6.0	< 0.001
Length of hospital stay (days)	13.6 ± 5.5	18.5 ± 7.6	< 0.001
Day of first flatus (postoperative day)	1.8 ± 1.1	2.3 ± 1.5	0.085

Data are presented as mean ± standard deviation

**Table 4:** Postoperative characteristics of patients without anastomotic leakage

Variables	No EPSBO (*n* = 357)	EPSBO (*n* = 38)	***P*** value
First ambulation (postoperative day)	2.0 ± 0.9	2.6 ± 1.5	0.025
Day of first meal (postoperative day)	3.0 ± 1.4	4.1 ± 3.6	0.379
Removal of drainage (days)	9.1 ± 2.5	11.53 ± 3.6	< 0.001
Length of hospital stay (days)	13.0 ± 4.2	16.8 ± 5.7	< 0.001
Day of first flatus (postoperative day)	1.8 ± 1.1	2.3 ± 1.7	0.097

Data are presented as mean ± standard deviation

## Discussion

EPSBO is the most frequent complication in colorectal surgery [12]. Previous studies reported an EPSBO incidence ranging from 4.5 to 12.8% [4]. The diagnosis of EPBO is complicated due to overlap between the symptoms and signs of postoperative patients and those of postoperative recovery, as well as the use of analgesics, which explains the wide range in incidence [5]. The findings of the current study showed an EPSBO incidence rate of 10.6%, which was relatively high. Therefore, it is important to explore risk factors for EPSBO. The current study retrospectively explored risk factors for EPSBO after right colectomy in 424 patients and constructed a nomogram to predict the risk of EPSBO after right colectomy. Previous studies have described many potential risk factors for EPSBO. After multivariate logistic regression analysis, the current study established a nomogram containing four risk factors. The C-statistic of this model showed a medium ability to predict the risk of EPSBO. The current study evaluated the risk of EPSBO based on this model to make individualized predictions of EPSBO and took postoperative preventive measures for patients who were assessed as high risk to reduce the burden on patients. Liang Wen Quan et al. [15] predicted prolonged posterior ileus and established a nomogram including preoperative albumin. Lv X et al. [16] established a model to predict bowel obstruction in preoperative colorectal cancer patients with clinical characteristics. The study subjects were mainly patients with preoperative bowel obstruction caused by malignant tumors and used a public database, which was different from our study. The current study is one of few studies focusing on the risk of EPSBO after right colectomy, and few studies have established nomograms for this disease. Therefore, new nomograms are urgently needed.

The findings of the current study showed that the probability of EPSBO was significantly higher in males than in females, which was consistent with previous studies [4, 6, 12, 13, 17]. Male patients may be more prone to EPSBO due to narrow pelvic space and high visceral fat content. However, since right colectomy falls under abdominal surgery, the degree of pelvic stenosis apparently has little effect on operation and postoperative complications. In addition, previous studies indicated that alcoholism and chronic lung disease increase the risk of EPSBO [5], and males account for the vast majority of these cases [18]. The current study did not collect data on alcoholism or chronic lung disease, although it can be postulated that the increased risk of EPSBO in males is due to multiple factors. Open surgery leads to an inflammatory response, whereas the use of laparoscopic surgery reduces abdominal injury and direct contact with intestines, resulting in a reduced inflammatory response and adhesion. This explains why open surgery is more prone to EPSBO and was an independent risk factor in the current study [19]. This is consistent with the findings of previous studies undertaken by Nakajima et al. [20], Masoomi Hossein et al. [5], and Eto Ken et al. [6]. Notably, some researchers believe that the incidence of EPSBO in the two operations is similar, which indicates that, although laparoscopic surgery reduces the inflammatory response caused by abdominal injury, laparoscopic surgery has a long operative time. However, the current study conducted subgroup analysis and found no significant difference in operative time between the two operations. Therefore, the current study suggests that open surgery is a risk factor for EPSBO in right colectomy, although technical variations among different surgeons may cause confounding bias.

A history of abdominal surgery increases the risk of EPSBO, which has been confirmed by previous studies [21, 22]. After multivariate analysis and controlling for confounding factors, a history of abdominal surgery was not an independent risk factor for EPSBO (*P* = 0.111). A long operative time was considered a very important independent risk factor in current and previous studies [4, 12, 14, 22]. Therefore, surgeons should complete the operation as soon as possible while ensuring surgical treatment to reduce complications caused by long operative times. The current study found a close relationship between preoperative serum albumin level and EPSBO, which indicates that poor systemic nutritional status increases the risk of EPSBO after right colectomy. This is consistent with the findings of Pan Chi et al. [14], who hypothesized that preoperative low albumin induced EPSBO. Hypoproteinemia leads to intestinal swelling, which results in a local inflammatory response in the small intestine and increases the risk of EPSBO. In addition, the current study showed that anastomotic leakage increased the risk of EPSBO. A retrospective analysis of the medical records of EPSBO patients showed that most EPSBOs occurred after anastomotic leakage. Therefore, the inflammatory response promotes the formation of intestinal obstruction by activating polymorphonuclear leukocytes [23].

Although extended resection (*P* = 0.365) and SMA as the medial boundary of lymph node dissection (*P* = 0.347) expanded the operation area, they did not significantly increase the risk of EPSBO. However, the dissection of lymph nodes on the SMA surface led to severe chylous ascites. Moreover, previous studies have shown that lymph node dissection damages autonomic nerves and leads to gastrointestinal dysfunction [10]. In clinical practice, we found a high incidence of chylous ascites after right colectomy; After completing this manuscript, we studied the risk factors of chylous ascites with curiosity and published it before the acceptance of this article [24]. Emergency surgery for patients with right colon cancer was generally due to complete intestinal obstruction, followed by intestinal perforation. Notably, emergency surgery was rarely performed for intestinal obstruction of right colon cancer in the study. Generally, endoscopic colonic stent implantation was performed first, which transformed emergency surgery into elective surgery, thereby increasing the possibility of laparoscopic surgery and speeding up postoperative rehabilitation. A previous study by Ji Woong BAE showed that 93% (13/14) of patients in the stent group underwent elective laparoscopic surgery [25].

The effect of EPSBO on postoperative rehabilitation of patients was explored in the current study by analyzing indicators of postoperative rehabilitation, including first ambulation time, day of first flatus, day of first meal, removal of drainage time, and length of hospital stay. We found that early out-of-bed activities can reduce the risk of EPSBO, which emphasizes the importance of early out-of-bed activities and verifies that ERAS can promote the recovery of gastrointestinal function [26, 27]. The findings showed that EPSBO significantly prolonged the drainage tube removal (9.4 ± 3.1 vs. 14.4 ± 6.0 *P* < 0.001) and hospital stay (13.6 ± 5.5 vs. 18.5 ± 7.6 *P* < 0.001), which greatly affected the rehabilitation of patients. Anastomotic leakage also significantly prolongs the length of hospital stay [28]. The findings of the current study showed that anastomotic leakage was a risk factor for EPSBO. Therefore, it was necessary to exclude patients with anastomotic leakage to more accurately show the effect of EPSBO on hospital stay. The findings showed that EPSBO still significantly prolonged hospital stay (13.0 ± 4.2 vs. 16.8 ± 5.7) after excluding patients with anastomotic leakage, indicating that EPSBO cannot be ignored. However, we found that the eating time and time to remove the drainage tube were late, which seemed inconsistent with the ERAS protocol. The main reason was the large time span of case collection, different competent doctors and patient safety considerations. In addition, we believe that EPSBO leads to the prolongation of drainage tube removal time. Patients with EPSBO are prone to abdominal pain, abdominal distention, or other symptoms that are necessary to eliminate anastomotic leakage, chylous ascites, and other life-threatening complications. On the other hand, EPSBO may lead to a large amount of plasma exudation and peritonitis, and it is therefore necessary to delay drainage tube removal.

Reoperation and mortality rates reported in the current study were lower than those of a study by Khrucharoen U et al. involving 184,660 people [29]. However, preventing the occurrence of EPSBO remains an important implication. Some of the risk factors identified in the current study can be controlled. For example, laparoscopic surgery should be used as much as possible, and it is recommended to convert emergency surgery into elective surgery through endoscopic technology to reduce the risk of EPSBO. During the operation, the intestine should be handled carefully, and the operation should be completed as soon as possible without performing optional maneuvers [4]. Hypoproteinemia should be corrected actively before the operation, and anastomotic leakage should be prevented. In addition, intraoperative preventive operations, including the use of starch-free gloves and flushing with normal saline below 37 °C, should be considered, which may reduce the incidence of small intestinal adhesion and obstruction [30]. For patients with high scores, antiadhesion drugs, such as sodium hyaluronate, can be given appropriately, or moxibustion can be used to reduce the risk of intestinal obstruction [31, 32]. In addition, more attention should be paid to the application of enhanced recovery after surgery (ERAS) to promote rapid rehabilitation [26, 27, 33]. For example, eating should be resumed as soon as possible, and the drainage tube should be removed early. We can even cooperate with information specialists to design an automatic computer application to better implement personalized management schemes for patients. At present, our department is trying to use this nomogram and is expected to share its practicability in the near future.

According to the latest study, we believe that CME has not proved inferior to traditional surgery in terms of feasibility and safety. And with the expansion of future research samples, this conclusion will be evaluated more accurately [34]. The current study constructed a novel nomogram based on the findings of multivariate logistic regression analysis, including male sex, open surgery, operative time, and anastomotic leakage. The nomogram provided surgeons with a predicted risk of EPSBO. This was established by perioperative clinical data, which can help surgeons formulate personalized preventive measures according to the degree of risk.

The current study had some limitations. First, due to the single-center retrospective design of the current study, potential selection bias could not be avoided. Second, since the current study undertook only internal verification, it was unclear whether the constructed nomogram has universal applicability. Finally, the operations were not performed by the same surgeon, and there may therefore be potential performance bias. The current study envisages prospective multicenter research in the future to improve the performance of the model.

## Conclusion

The current study explored risk factors for EPSBO after right colectomy through intergroup comparisons and obtained independent risk factors for EPSBO through multivariate logistic regression. Therefore, a nomogram was constructed to predict EPSBO risk, which has good predictive ability, can accurately provide individualized prediction of EPSBO, and plays an active role in patient management.

## Data Availability

The datasets used and/or analyzed during the current study are available from the corresponding author on reasonable request.

## Abbreviations

EPSBO: Early postoperative small bowel obstruction
C-statistic: Concordance statistic
ROC: Receiver operating characteristic curve
AUC: Area under curve
ASA: American Society of Anesthesiologists
OR: Odds ratio
CI: Confidence interval
BMI: Body mass index
SMA: Superior mesenteric artery
SMV: Superior mesenteric vein
NLR: Neutrophil-lymphocyte ratio
ERAS: Enhanced recovery after surgery
