# Correction to: Novel nomogram for predicting risk of early postoperative small bowel obstruction after right colectomy for cancer

**DOI:** 10.1186/s12957-022-02499-0

**Published:** 2022-03-10

**Authors:** Huida Zheng, Yurong Liu, Zhenze Chen, Yafeng Sun, Jianhua Xu

**Affiliations:** Department of Gastrointestinal Surgery, The Second Afliated Hospital of Fujian Medical University, No. 950 Donghai Street, Fengze District, Quanzhou, 362000 Fujian Province China

**Correction to: World J Surg Oncol 20, 19 (2022)**

**https://doi.org/10.1186/s12957-022-02489-2**

Following the publication of the original article [1], the author reported that Figure 1 was not updated as per correction and that Table 1 corrections were incorrectly carried out.

The original article has been updated.
